# The Route of Biogenic Amines in Alcoholic Beverages: A Focus on Wine

**DOI:** 10.3390/foods15091457

**Published:** 2026-04-22

**Authors:** Luigi Esposito, Andrea Piva, Dino Mastrocola, Maria Martuscelli

**Affiliations:** Department of Bioscience and Technology for Food, Agriculture and Environment, University of Teramo, via Balzarini 1, 64100 Teramo, Italy; lesposito2@unite.it (L.E.); apiva@unite.it (A.P.); dmastrocola@unite.it (D.M.)

**Keywords:** biogenic amines, histamine, tyramine, wine, human health, intolerance, allergy

## Abstract

Biogenic amines (BAs) are important qualitative indicators of quality, as they are produced by specific microbial strains and can therefore reflect the activity of specific spoilage organisms (SSOs). Their presence in food, including wine, provides valuable information on processing conditions, hygiene practices and storage management throughout the production chain. In wine, the accumulation of BAs—particularly histamine, tyramine and putrescine—is mainly associated with microbial activity during fermentation, especially malolactic fermentation, and may pose potential risks to consumer health. Despite the recognized toxicological relevance of BAs, current European Union (EU) regulations only establish limits for histamine in certain fish products, with no specific legal thresholds defined for wine. However, growing evidence on the interactions and adverse effects of BAs highlights the need to better address their occurrence in wine and to improve consumer awareness regarding safety and quality aspects. In addition to safety concerns, the implementation of good hygiene and manufacturing practices across the entire production process plays a crucial role in controlling BA levels in the final product. These factors, together with the intrinsic characteristics of wine, may influence consumer perception and choice, integrating aspects of health, production methods and product quality. Recent findings suggest a shift in perspective, where BAs are not only considered risk markers but also useful indicators for assessing wine quality and enhancing consumer safety.

## 1. Biogenic Amines in Food and Beverages

BAs are low-molecular-weight nitrogenous compounds (mainly polar bases) with different structures depending on the number of aminic groups in the final product [1].

**Figure 1:**
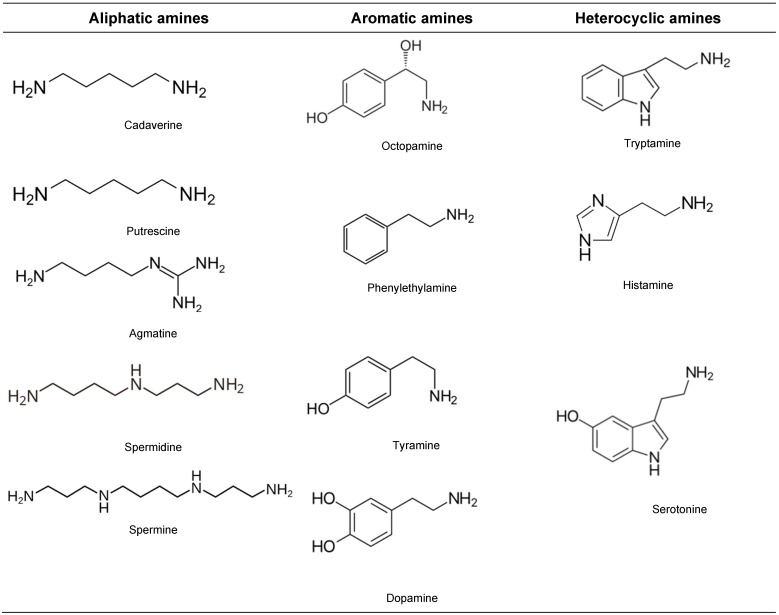
Classification of BAs based on their chemical structure.

Figure 1 and Table 1 show the main classification systems adopted for BAs (those most found in foods and beverages). These biocompounds are naturally present in microorganisms, plants and animal organisms’ cells, playing important physiological roles, as signaling molecules under stress conditions, during cell renewal, growth, tissue protection, etc. Their occurrence may depend on reactions of amination or transamination of aldehydes and ketones [2], but it is mainly related to decarboxylation reactions converting amino acids to BAs [3]. This biochemical pathway relies on the activity of a class of enzymes, decarboxylases, present in several strains of microorganisms (bacteria, yeasts and molds) able to catalyze the cut of an aminic group from a free aminoacid giving a corresponding biogenic amine and a molecule of CO_2_ [4].

Due to BAs’ ubiquity, and the strict interconnection with microbial metabolism, these bio-compounds are largely present in foods and beverages, rarely representing a consistent hazard. In 2011, the European Food Safety Authority (EFSA) [5] conducted a qualitative analysis on the hazard represented by fermented foods from all over Europe, pushing researchers to investigate in more depth the relationships between food and BA contents. The collection of data from EFSA indicated the amounts of BAs, in several foods and beverages, questioning on their possible harmful on human beings; also creating a large database. During recent years, different studies have approached BAs, trying to collate all the updates with a view to understanding their real impact on human life. One of the hard parts of BA comprehension is their identification in food matrices. Even if various techniques are reported and discussed in the literature [6,7,8], high-performance liquid chromatography (HPLC) still represents the reference method of official laboratory analysis [9,10,11]. Moreover, innovative methods [12,13,14] are used to reach lower detection limits and perform analytical procedures with fewer sample processing steps [15].

The present review focuses on BA presence in wine for different reasons. There is an urgency to correctly inform consumers, as previously detailed, about BAs’ natural presence in grapes, their change during manufacturing and the essential fermentation step vital for winemaking, but also for BA production. This is also important in consideration of the social value attributed to wine, and alcoholic beverages in general, considering a combination effect in meals, thus contributing to maintaining consumers’ awareness of potential risks, especially for sensitive individuals.

## 2. Controversial Behavior of BAs on Human Health

The impact on health due to diet is a vast research field, and how biogenic amines (BAs) interact with humans represents a trend-topic in recent years [16].

BAs are essential for the body’s good functioning and are even endogenously produced; they are the bases of several hormones and are also involved in a myriad of biological pathways such as thermoregulation, gastric acid secretion and signaling activity, among others [17].

Endogenous amines include neurotransmitters synthetized de novo in the body and transmitted locally or through the circulatory system [18,19].

Differentiation must also be drawn between those BAs highly involved in neurodegenerative conditions associated with dopamine, serotonin, norepinephrine and epinephrine that are commonly less or not present in this form in foods, but endogenously produced [20]. Moreover, recently, the focus has been on salsolinol (derived from dopamine and potentially neurotoxic) [21]. In contrast, exogenic amines are naturally occurring anti-nutritional factors found in both processed and raw foods when spoilage or uncontrolled storage occurs [22].

In normal conditions (homeostasis and absence of specific medical conditions), BAs are easily managed by the human metabolism through deputed enzymes located in the intestine, named Mono and Di-Amino Oxidase (MAO and DAO, respectively). Generally, the detoxification of BAs introduced with foods and beverages is fast and painless, but several conditions may arise [23,24], with symptoms from mild to very severe. Respiratory, gastrointestinal and dermatological problems, as well as headaches and migraines, are the principal symptoms of exposure to BAs (Table 2).

The vast range of body targets leading to the onset of several discomforts represent a problem for correct diagnosis [16], and in fact, nowadays, uncertainties remain about the responses that BAs can provoke in humans.

Mainly uncontrolled HIS levels generate communitarian alerts. In Europe, alerts about HIS, other governed compounds and contaminants are controlled by the RASFF system. The European Legislation, by means of Reg. CE 2073/2005, Reg. CE 1441/2007 and Reg. UE 365/2010 [25,26,27], has set limits just for HIS since the recognition of the syndrome “scombroid fish poisoning”, which depends on the consumption of fishery-origin products [28].

The Joint FAO/WHO Expert Committee [29] described BA intolerance as separate from the well-known HIS intoxication, also highlighting that they may affect individuals who do not commonly eat fishery products or fermented foods [30]. More recently, TYR was also considered responsible for similar conditions giving rise to another syndrome: the so-called “cheese reaction” [31,32]. TYR is typically commonly found in cheeses of all origins due to the decarboxylation activity of LAB, *Pseudomonadaceae*, *Enterobacteriaceae* and *Micrococcacee* [33].

As del Rio et al. [34] and Sanchez-Perez et al. [23] found, the cytotoxicity of TYR is enhanced by HIS even when it is contained at low levels. Moreover, TYR and HIS have a synergistic role, resulting in a boosting effect on consumers who may experience strong migraines, itching, nausea, pressure disorders and other symptoms. The occurrence of the “cheese reaction” is more dangerous for patients treated with Monoamine Oxidase Inhibitors (MAOIs). These drugs are successfully used to treat depression, panic and social anxiety disorders, among others [35], but can badly interact with TYR, leading to hypertensive crisis [36].

The consumption of wine is often associated with adverse reactions such as headaches, migraines and allergy-like symptoms, which are increasingly linked to the presence of BAs, including HIS, TYR and PUT (Table 2) [23,37]. Moreover, it is also well-documented how much CAD and PUT, at concentrations five times higher than HIS, enhance its toxicity [38]. Other BAs receive reduced attention, lacking correlated medical conditions because of their limited inclusion in foods and beverages. Recently, it was understood how other BAs increase the severity of manifestations, even when ingested in small amounts, in quantities possibly present in foods [23].

On this note, recent publications about the impact of other BAs, such as ethanolamine, ethylamine and methylamine, on health must be underlined, including their involvement in HIS and TYR toxicity [37].

In any event, no actions were undertaken to better understand how BAs can affect human health in everyday food consumption.

Researchers find it difficult to propose thresholds for safeguarding people’s health, in relation to BAs, while maintaining food safety and quality [39]. On one hand, it is difficult defining BAs’ hazardousness and the connected risk for health; on the other hand, anyone may experience one or more symptoms linked to BA exposure, have changing individual sensitivity to them and experience the acute manifestation of any health problem. Thus, according to the data available and the registered cases, BAs’ possible harmful effects on a segment of the population is unknown and unpredictable [9,36,40,41,42,43,44].

Primarily, it is difficult to establish universal thresholds because of the tremendous differences among individuals, concerning personal medical conditions, genetic impairments of MAO and DAO enzymes, drug treatments, individual sensitivity, dietary patterns and food choices. These two conditions have still not received strong consideration by the scientific community due to the difficulty of obtaining realistic data about people’s habits. Furthermore, even regarding BA management, what we eat and its quantity, may impact the possible BA effect. It is recognized, for example, that one of the main inhibitors of DAO activity is alcohol [45,46], and nowadays, one of the primary treatments for people experiencing symptoms of BA exposure is an HIS-free diet, excluding foods as specific fishes, as well as fermented products including wine, chocolate and others [23,47]. Also, important advancements are carried out for the understanding of MAO and DAO functioning for giving most affordable and eco-friendly ways of producing drugs and supplements for people with this condition [48,49].

Even low amounts of HIS in several ingredients and meals can trigger negative reactions in those subjects with a deficiency in DAO, the enzyme responsible for HIS detoxification [23,46]. Therefore, people already suffering from high blood pressure, or at risk for cardiovascular diseases, are more sensitive to HIS poisoning, having developed a genetic DAO deficiency [50].

Even the great effort undertaken by EFSA in 2011 [5] to have sufficient data to set limits did not mention how diets and habits in food consumption can have a role in the health of both at-risk and not-at-risk people. Unfortunately, despite all the efforts to understand how the quality of foods and beverages is also affected by the occurrence of biogenic amines, too little attention has been paid to making consumers aware of this issue.

## 3. Biogenic Amines and Process Dynamics of Wine

**Figure 2:**
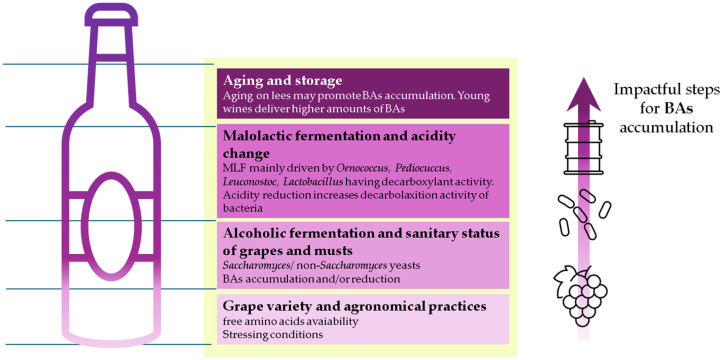
Main steps allowing BA presence in wine.

The following section analyses the predominant conditions favoring BA presence in wine (Figure 2). Those on which we focused more are as follows: free amino acid presence, environmental factors, microbial community, environmental conditions, ethanol presence and other technological factors.

Wine represents an ideal substrate for BA formation due to a combination of its chemical composition, microbial dynamics and processing conditions. Additionally, as outlined in the technical manuals purposed by the International Organization of Vine and Wine (OIV) [51], it is crucial to maintain high levels of hygiene and manufacturing practices during fermentation, as is fundamental to have notions about the role of agricultural practices on grapevines.

Considering what will be discussed below, it is very difficult to precisely attribute the primary responsibility for BA accumulation in wine, because many factors are involved (Table 3).

### 3.1. Grape Variety and Agronomical Practice

According to many authors, one of the primary factors is the availability of free amino acids (FAAs) in grapes, serving as precursors for BAs [52]. The presence of FAAs is greatly variable, being strongly associated with the grape’s genetic information and climatic factors that change on a yearly basis [94]. Amino acids are abundant in grapes because they play essential roles in the plant’s metabolism, particularly during berry development and ripening. Grapevines synthesize amino acids as part of their nitrogen metabolism, which is crucial for protein synthesis, growth and responses to environmental stress [95,96]. This abundance is primarily due to several interconnected metabolic processes. First, amino acids act as intermediates in primary metabolic pathways such as the Krebs cycle and glycolysis, supporting both energy production and the biosynthesis of essential compounds. Additionally, grapevines assimilate nitrogen from the soil, converting it into amino acids like glutamine, glutamate and asparagine, which serve as key nitrogen carriers within the plant [97]. During the ripening process, protein degradation and nitrogen mobilization further contribute to the accumulation of free amino acids. This not only supports the plant’s metabolic needs but also leads to the formation of precursors for flavor and aroma compounds, influencing the sensory profile of grapes and wine. Moreover, under environmental stresses such as drought or high temperatures, grapes increase the production of specific amino acids like proline. Proline acts as an osmoprotectant, helping to stabilize cellular structures and maintain osmotic balance under adverse conditions [98].

The concentration and composition of amino acids vary significantly among different grape varieties due to genetic differences, environmental conditions and viticultural practices [94]. In white grape varieties such as Chardonnay and Sauvignon Blanc, amino acids like arginine and proline are often present in higher concentrations. Riesling, on the other hand, tends to have elevated levels of glutamate and aspartate, which contribute to its distinctive aromatic profile. Among red grape varieties, Cabernet sauvignon and Merlot generally accumulate more proline, particularly under water stress conditions, as well as arginine, which plays an important role in yeast metabolism during fermentation. Pinot noir typically exhibits a balanced amino acid profile, with notable amounts of glutamine and alanine. Some varieties, such as Tempranillo, Syrah and Grenache, are particularly rich in arginine, which can significantly influence fermentation dynamics since many yeast strains preferentially metabolize this amino acid [53,54].

Environmental factors, including soil nitrogen availability, climate and vineyard management practices, also have a profound effect on amino acid levels. For example, nitrogen fertilization tends to increase the overall amino acid content in grapes, while water stress conditions often lead to elevated proline levels due to its role in stress response. This complex interplay between genetic and environmental factors ultimately shapes the amino acid profile of grapes, with important implications for both grape physiology and wine production. Additionally, vineyard management practices, including soil fertilization, nitrogen supplementation and grape variety selection, significantly influence the amino acid profile of the grapes [55].

This condition makes it difficult to estimate a constant level of BAs in wines on a quantitative and qualitative scale. On this note, Yañez et al. [99] found that, generally, organically obtained wines have lower BA levels; Cravero [56] screened the characteristics of biodynamic wines that, on one hand, should be more susceptible to BA accumulation for specific winemaking procedures such as spontaneous fermentation, and on the other, by following strict procedures during grape obtention, less FAAs should be available. Few references are available on this. Additionally, Tassoni et al. [57] and Costantini et al. [58] identified no significative differences in wines obtained from different agricultural practices, including biodynamic ones.

Other FAAs, such as histidine, tyrosine, ornithine and lysine (directly linked to dietary BA production), are abundant in grape must and wine, providing the necessary substrates for BA synthesis. Their concentration increases during grape ripening and is further augmented by yeast autolysis during fermentation. The increased availability of FAAs may augment the capabilities of those decarboxylase-positive strains in producing BAs, but some studies did not find this direct correlation [100]. On the contrary, Hernandez-Macias et al. [101] identified how Cava lees are a useful ingredient for mitigating BA production in other fermented foods.

### 3.2. Microbial Community in Winemaking

The microbial community involved in winemaking greatly define final BA levels in wine. Producing wines using selected strains is an established practice; strains are even validated for BA production activity. Spontaneous fermentations, often used in traditional or natural winemaking, involve a wide range of microorganisms, including wild yeasts and lactic acid bacteria (LAB), many of which possess amino acid decarboxylase activity, leading to BA formation. Modulating fermentation dynamics may also have a key role in reducing nitrogen substrates for BA production. A recent work of Tan et al. [59] underlines the role of non-*Saccaromyces* yeasts on BA mitigation while producing desired sensory characteristics. Authors have found *Schizosaccharomyces pombe* and *Lachancea thermotoleran* to be very helpful in maintaining safe BA levels in wines. These discoveries are particularly important in the light of other fermentation steps to which wine is subjected, such as malolactic fermentation (MLF). This is a common secondary fermentation process, playing a particularly important role in this context. As recently reviewed by Moreira et al. [60], plenty of LAB species, such as *O. oeni* and *Lactobacillus* spp., are known to decarboxylate amino acids, significantly increasing levels of HIS, TYR and PUT. These authors have also highlighted how much the geographical provenience of the grapes shape the potential of the obtained wines to deliver the BAs HIS and TYR, which carry microbial heterogeneity. Moreover, inadequate hygiene during winemaking can lead to contamination with spoilage bacteria like *Pediococcus* and *Enterococcus* spp., which are also capable of producing BAs.

Furthermore, in addition to the use of selected non-BA-producing starter cultures, the reduction in these compounds in fermented products can also be enhanced by using bacteria capable of degrading them [102,103,104,105]. Strains belonging to the *Lactobacillus* and *Pediococcus* groups were found to have the greatest BA-degrading activity in wine [61,62], suggesting this ability as a new criterion for selecting winery starter cultures. Other authors reported on enzymes isolated from strains of *L. plantarum* and *P. acidilactici*, and identified multicopper oxidases able to degrade BAs relevant in wine (PUT, HIS and TYR) [63,106].

The environmental conditions during winemaking further promote BA formation. Wine’s acidic pH, typically ranging from 3.0 to 4.0, creates an environment that, while inhibitory to many pathogens, is optimal for certain LAB and spoilage bacteria with decarboxylase activity. As seen by Kushnereva [64], a more acidic pH limits BA stability in wine, while values nearing a pH of 4.0 allow BA stability; this is one reason why red wines are commonly richer in BAs. By nature, BAs are inhibited by acidic environments [41]; thus, LABs limit BA accumulation in foods by producing lactic acid in the matrix. Other authors remark that the optimal values for histidine, tyrosine and ornithine decarboxylase activation are 4.8, 5.0 and 5.8, respectively [47,65,66]. Perestrelo et al. demonstrated the direct correlation between higher pH and increased HIS levels in wine [67].

As for pH, the presence of ethanol, another characteristic compound of wine, has a dual role. While it can inhibit the growth of some microorganisms, it also selects for ethanol-tolerant BA-producing bacteria. Studies have demonstrated that ethanol concentrations, such as those found in wine, can stimulate the growth of LAB and other bacteria known for BA production. Bover-Cid et al. [69] highlighted that LAB, especially strains of *Lactobacillus* and *Enterococcus*, can be more active in ethanol-rich environments, as they often possess ethanol-tolerance mechanisms. Ethanol can act as a stressor for microorganisms, which may induce the production of amino acid decarboxylases as part of their stress response. The presence of ethanol may also influence the availability of precursor amino acids, which are substrates for BA formation. Serrazanetti et al. [70] discussed how ethanol induces the expression of decarboxylases in LAB and other microorganisms. The presence of ethanol can affect the regulation of these enzymes, thus facilitating increased BA production; ethanol alters the permeability of microbial cell membranes, potentially increasing the uptake of amino acids and promoting the decarboxylation process that leads to BA formation. Ingram [107] showed that the ethanol content in wine can promote the uptake of these amino acids by bacteria, thus enhancing BA production. Ethanol also affects the overall metabolic shifts in the microbial community. It may inhibit some fermentation pathways while promoting others that favor the production of BAs. Ethanol enhances the conditions under which amino acid decarboxylation can occur more readily, especially under low-oxygen wine fermentation conditions. Rosi et al. [71] reviewed how ethanol influences microbial metabolism during wine fermentation. They noted that ethanol, especially in combination with low oxygen availability, favors pathways that lead to increased BA concentrations in the final product. Ethanol plays a significant role in modifying the microbial environment and the enzymatic activities involved in the synthesis of BAs. Recently, Míšková et al. [72] analyzed 232 wine samples from Central Europe, focusing on the presence of eight BAs; they found that ethanol content influenced microbial activity and BA levels.

Temperature exerts a significant influence on the presence and accumulation of biogenic amines (BAs) in wines, as it directly affects microbial growth, enzymatic activity and the progression of both alcoholic and malolactic fermentations. Elevated temperatures during grape processing, fermentationor wine storage can promote the proliferation of LAB, such as *Oenococcus oeni*, *Lactobacillus* spp. and *Pediococcus* spp., which possess amino acid decarboxylase activity responsible for the synthesis of HIS, TYR, PUT and other BAs [61,73,74]. In particular, temperatures above 18–20 °C during malolactic fermentation tend to enhance LAB metabolic activity, increasing the risk of BA formation, especially when accompanied by poor hygienic practices or nutrient imbalances in the wine matrix [75,76,77]. Moreover, temperature fluctuations during wine storage can lead to microbial instability, favoring the growth of spoilage microorganisms capable of producing BAs, thereby compromising wine safety and quality [62,78]. In contrast, maintaining low and stable temperatures during both fermentation and storage (e.g., below 15 °C) can reduce bacterial activity and decarboxylase enzyme expression, thus minimizing BA production [79,80]. Therefore, temperature control throughout winemaking and storage is a fundamental preventive measure to limit BA formation and ensure the production of wines with acceptable safety and sensory profiles. Several studies have investigated how different temperatures during wine production influence the accumulation of BAs. One study examined the effects of temperature, alcohol content and amino acid supplementation on the levels of TYR, HIS, 2-phenylethylamine and tryptamine during winemaking. The researchers found that TYR synthesis was primarily affected by temperature and alcohol content, while tryptamine synthesis depended mainly on temperature. Interestingly, amino acid supplementation did not consistently lead to higher BA concentrations, except for HIS, which increased with the addition of specific amino acids [108]. Another study focused on the impact of adding complex commercial yeast and bacterial nutrients, as well as different malolactic fermentation (MLF) inoculation scenarios, on BA production in wine. The results indicated that the addition of complex nutrients could potentially increase HIS concentrations. Regarding MLF inoculation strategies, co-inoculation resulted in lower BA concentrations in Shiraz wine but higher concentrations in Pinot noir aged wine, suggesting that the effect of inoculation timing on BA levels may vary depending on the grape cultivar [109].

The timing and method of MLF initiation matter; early co-inoculation with selected LAB strains often results in lower BA levels compared to spontaneous or late inoculation, which allows spoilage bacteria to flourish [81,82].

### 3.3. Winemaking Process

In a study examining 65 wines from the Abruzzo region, Martuscelli et al. [83] investigated the effect of agronomic and oenological factors on BA content. They found that red wines had higher total BA levels (19.3 ± 12.8 mg/L) compared to rosé (9.20 ± 6.34 mg/L) and white wines (7.67 ± 3.84 mg/L). These differences were attributed to distinct winemaking processes and microbial activity associated with each wine type. In fact, red wines tend to contain higher BA levels compared to white and rosé wines due to extended maceration and malolactic fermentation, increasing the likelihood of triggering the described adverse effects [110].

Sulfur dioxide (SO_2_) additions and other antimicrobial agents help suppress undesirable bacterial populations [111]; however, some authors report that low SO_2_ concentrations or its absence in natural or minimal-intervention winemaking may heighten BA risk [78,110]; meanwhile, in other studies, no statistical differences were found between BAs in wine with or without added sulfur dioxide [65]. Aging on lees (sur lie) can either increase or decrease BA levels depending on microbial stability; while autolysis releases nutrients that could favor microbial activity, lees aging under reductive conditions often inhibits spoilage bacteria [112].

Recent studies have further elucidated how various winemaking techniques influence the presence of BAs in wine. Maceration practices, for instance, have been shown to significantly impact BA formation. Semjon et al. [113], in a study on Slovak Tokaj wines, demonstrated that maceration, especially when combined with the addition of *Saccharomyces cerevisiae* cultures, led to BA formation; the research indicates that the addition of complex commercial yeast and bacterial nutrients can potentially increase HIS concentrations in wine. This effect could be related to the increased transfer of amino acids to the wine-juice (following contact with the grape skin), which in turn determines a greater availability of the substrate for BA formation. A study by Smit et al. [84] showed that the relationship between maceration and biogenic amine formation is not solely dependent on the extent of skin contact, but rather on its timing and its interaction with malolactic fermentation. In particular, although cold pre-fermentative maceration initially promotes the extraction of amino acid precursors, it ultimately leads to lower biogenic amine concentrations in the finished wines compared to conventional, extendedor even no-skin-contact treatments. Conversely, wines subjected to conventional and extended maceration, as well as those produced without skin contact, showed higher final levels of biogenic amines after malolactic fermentation. These findings suggest that early-stage maceration may modulate precursor availability and/or microbial activity in a way that limits decarboxylation during malolactic fermentation, which appears to be the critical phase for biogenic amine accumulation.

Additionally, aging on lees can influence BA content due to the release of amino acids from yeast autolysis, which serve as precursors for BA synthesis. A study examining the evolution of amino acids during 12 to 18 months of lees aging found that certain commercial maturation products increased the levels of essential amino acids, potentially leading to higher BA formation [85].

Among winemaking processes, filtration and clarification practices are pivotal in controlling BA-forming bacteria; inadequate filtration, especially before bottling, can permit bacterial survival and post-bottling BA production [62]. Conversely, sterile filtration effectively reduces microbial loads, minimizing the risk of BA synthesis during storage; moreover, effective filtration methods, such as the use of mesoporous silica materials functionalized with phosphonic and sulfonic acids, have been shown to remove significant amounts of BAs, including HIS and PUT, thereby enhancing wine safety and quality [86].

Implementing good winemaking practices is vital to minimizing BA production. Managing must and wine pH, stabilizing with sulfur dioxide or biological alternatives, and using malolactic bacteria strains screened for the absence of BA-producing genes are effective strategies to control BA levels in wine.

The production of wine under conventional, organic, biodynamic and natural approaches entails distinct viticultural and winemaking practices that significantly impact microbial ecology and biochemical transformations influencing BA levels. In conventional viticulture, synthetic pesticides, fungicides and fertilizers are extensively employed to control pests and optimize grape yield and quality, resulting in grapes with fewer microbial contaminants [87]. During winemaking, SO_2_ is routinely used as an antimicrobial and antioxidant agent, coupled with strict temperature control, filtration and the inoculation of selected *Saccharomyces cerevisiae* yeasts, which collectively reduce the proliferation of BA-producing bacteria such LAB [88,89].

Conversely, organic viticulture prohibits synthetic agrochemicals, relying instead on natural treatments (e.g., copper, sulfur and plant-based solutions), which can result in grapes with a more diverse microbiota and potentially higher initial bacterial loads [90]. In organic winemaking, the reduced application of SO_2_ and increased frequency of spontaneous fermentations amplify the risk of BA formation, particularly HIS, PUT and TYR [91,114]. Biodynamic viticulture, which aligns with organic principles but integrates lunar cycles and the use of specific composts and plant extracts, promotes soil health and biodiversity; however, these practices similarly favor microbial heterogeneity, increasing the potential for BA-producing bacteria on grapes and during vinification [89]. Natural winemaking, often associated with biodynamic or organic grape cultivation, represents the least interventionist approach. It typically involves spontaneous fermentation with indigenous yeasts and minimal to zero SO_2_ additions, while filtration and clarification steps are often omitted. This results in an extended microbial presence throughout vinification and aging, substantially heightening the likelihood of BA accumulation [92,115]. Studies comparing wine styles have revealed that natural and organic wines frequently exhibit higher BA levels than their conventional counterparts, underscoring the need for careful microbial management to balance wine authenticity and safety [87,110,116]. The interplay between grape cultivation and winemaking choices thus emerges as a critical determinant of BA content, making it imperative for producers of organic, biodynamic and natural wines to adopt strategies that mitigate microbial spoilage while preserving the desired sensory and environmental qualities of their wines.

The occurrence of HIS and TYR in wine could be a constant, as reported about rosé and white wines produced in Spain (Cataluña) [115] or red wines from Chile, California, various European countries (France, Germany, Spain), as well as in Italy [117,118,119]; even the presence of amines could be associated with the geographical origin and the cultivar [120]. On this note, one study indicates that the apparent increase in biogenic amines in white wines during storage is mainly due to their low initial concentrations and more homogeneous amine profile, which make slight variations over time more detectable. However, storage does not significantly affect the total biogenic amine content, which is primarily determined by the levels present at the time of bottle opening [121]. Moreover, aging and storage play a role too (Figure 2), and the use of materials such as wooden barrels may contribute to the evolution of BAs; wines aged in oak barrels or stored under improper conditions can accumulate higher BA levels due to microbial activity and amino acid transformations [53].

## 4. Wine Allergy, Wine Intolerance and Biogenic Amines Implication

According to data presented in 2018 by the World Health Organization (WHO) [122], more than two billon people worldwide consume alcoholic beverages, confirming that ethanol is present in all diets. When considering wine intolerance and allergy, ethanol represents the main triggering agent for these conditions. Additionally, HIS and other biogenic amines, ethanol, acetaldehyde, acetic acid, flavonoids and sulfites are causative agents of intolerance reactions (pseudoallergic reactions) to wine [123].

Cerutti et al. [124], by means of a questionnaire, observed a high prevalence (6% of 901 respondents) of intolerance to wine in Italy, with no differences among the type of wine (red or white), highlighting that mainly young people and women may be more exposed to these phenomena. Other similar studies have shown a lower prevalence of wine intolerance in Germany (3.2%), but higher in Denmark (13%), reporting that red wine could be the main cause for hypersensitivity symptoms, as well as spirits [125].

Patients with hypersensitivity reactions to white or red wine always assume to be suffering from an “allergy”, but an important distinction should be made between immunologically mediated wine allergy and wine intolerance (Type 1 allergic reactions, no specific IgE are detected in the serum).

Among BAs, HIS is implicated in wine intolerance symptoms resembling allergic responses, such as flushing, palpitations, hypotension, gastrointestinal disturbances and respiratory issues [45]. Menne et al. [126] reported that histamine intolerance was triggered in susceptible individual by the intake of 4 mg of HIS (due to consumption of 200 mL of wine with 20 mg/L of histamine content). Individuals with reduced DAO enzyme activity, responsible for HIS degradation, are more susceptible to HIS intolerance, with wine being a frequent trigger due to its BA content and ethanol, which further inhibits DAO activity [48]. Migraines and vascular headaches are also linked to TYR, which can provoke hypertensive crises in susceptible individuals by stimulating norepinephrine release and increasing blood pressure [36,127,128]. Although SO_2_ and other wine components like tannins, flavonoids and alcohol have been traditionally blamed for wine intolerance and headaches, growing evidence emphasizes the role of BAs as key contributors, especially in individuals with impaired amine metabolism [46,121,129]. Acetaldehyde, too, an intermediate metabolite of ethanol oxidation, has been shown to exacerbate HIS-related symptoms. Acetaldehyde impairs aldehyde dehydrogenase (ALDH), an enzyme involved in the breakdown of acetaldehyde and other aldehydes, including some products of amine oxidation [76]. The accumulation of acetaldehyde may also enhance vasodilation and contribute to the so-called “Asian flush syndrome,” in which individuals with ALDH2 gene polymorphisms experience heightened alcohol intolerance symptoms [130]. Furthermore, acetaldehyde can directly sensitize sensory nerves and amplify HIS-mediated effects, worsening alcohol-induced headaches and flushing [3].

The tremendous and known impact that ethanol has on health influences wine consumption decision-making, contributing both to choosing between different wines and to the frequency and quantity of consumption. Deroover et al. [131] reviewed a vast array of literature on this topic; on the basis of fifty-two publications included in a final summary, these authors concluded that a controversial role of health resulted in wine consumption decision-making. In their synthesis, they reported that consumers generally perceive wine as healthier than other alcoholic beverages; in addition, they observed that red wine is perceived to be healthier than white wine. The overview covers measured outcomes related to pregnancy risk, calorie content, cancer risk and the effects of excess alcohol associated with wine consumption.

However, no study reports on opinions expressed about the risk associated with BAs in wine, because the scientific community has not yet brought this issue to the attention of consumers; this is also likely due to the lack of relevant regulations. In contrast, consumers have a clear perception of the health impact of sulfites in wine, so they often associate them with headaches; moreover, headache syndrome sufferers are willing to pay a premium for sulfite-free wine [132].

The presence of BAs is subtle in wine, as in other foods, because it does not alter the sensory characteristics of the product; in fact, only well-trained wine assessors can be able to identify elevated HIS concentrations [133].

Furthermore, the lack of legislation about BA limits in wines makes it difficult to correctly judge the imports and exports of these products, so Switzerland accepts those that do not exceed 10 mg/L of HIS, and Holland rejects wines containing more than 3.5 mg/L of HIS; moreover, the upper limit of histamine in wine is set at 8 mg/L in France, 5–6 mg/L in Belgium and 2 mg/L in Germany [72].

In contrast with the OIV guidelines [51], the available literature data demonstrate that a considerable number of wines (mostly red) are commonly placed on the European market with concerning BA levels. In detail, in the study of Jastrzębska et al. [118], 53% of the sampled wines purchased in Polish local markets had HIS contents higher than 8 mg/L; similarly, Restuccia et al. [134] registered an increasing trend of total BAs (from 23 to 46 mg/L) in Calabrian red wines from spontaneous fermentation from 2011 to 2018. Moreover, Perestrelo et al. [67] showed a positive trend for the occurrence of HIS in Verdelho wines from different vintages (2011–2015). Recently, Míšková et al. confirmed a prevalence of HIS occurrence among red wines from Central Europe [72]; in this study, 9% of 35 investigated red wines had HIS concentrations above 10 mg/L, while two samples also had PUT and TYR at alarming levels for sensitive individuals.

Since the final concentration of BAs in wine is the result of a complex balance between formation and degradation reactions, it is difficult to ensure processes designed for obtaining products with low or zero amine contents. Additionally, data concerning the absence of HIS and/or TYR in different wines are reported in many studies carried out in the last thirty years and are enclosed in Table 4. All these studies were carried out by determining the mean of official HPLC methods with an ultraviolet detector (HPLC-UV) or fluorescence detector (HPLC-FL), or liquid chromatography coupled with an electrospray ionization mass spectrometry (LC-ESI-MS) detection system. In particular, the absence of HIS and TYR resulted in white wines in 100% of those tested in Sardinia, in 95% among samples from the Abruzzo region, and in more than 90% of Croatian wines (see Table 4). Comuzzo et al. [135] highlighted that BAs may be problematic for organic products in relationship to a bad management of malolactic fermentation, high pH and volatile acidities. As a matter of fact, only 16% of 105 European investigated samples were TYR- and HIS-free. Additionally, it is very difficult to obtain wines without BAs, despite the correct management of critical technological factors such as the fermentation processes [52].

Drafting general considerations about wine safety and BA occurrence can lead to controversial information due to wine diversity and BA presence.

On one hand, all the information revised and discussed in previous sections must be considered; on the other, encouraging data about safety and HIS and TYR absence are reported. As seen by Kokole et al. [136], labeling strategies on alcohol health warning labels may enhance consumers consciousness, while promoting healthier diets. So, a possible path to follow is to emphasize in labels the HIS and TYR absence, or their limited presence. This approach could simultaneously communicate wine safety and guide the consumer towards an informed choice, without creating panic or unjustified refusal. Wine industry operators who already produce BA-safe products could adopt this issue as a development strategy to increase their market share, increase market size and profitability, and remain competitive in the market.

In the absence of an adequate regulation, to encourage producers to voluntarily adopt the control of biogenic amines as a quality standard, it is extremely important for wineries to identify a rapid, simple and highly sensitive analytical procedure.

Many scholars have investigated alternative strategies to the official HPLC method, to perform and validate methods for the determination of BAs in food and beverages. For the determination of HIS in wines, Marcobal et al. [100] proposed applying the enzyme-linked immunosorbent assay (ELISA) method and validated it by comparison with HPLC analysis; these authors found a good correlation between ELISA and HPLC methods. The presence of HIS in wine by means of the ELISA method allows the visual detection of HIS in only four minutes. This procedure could be a valid tool for controlling wine production in-line, before bottling, without any specific requirement from operators; the reaction is based on a color shift detecting and quantifying HIS.

As reported in a recent overview by Pereira et al. [137], biosensors are highly sensitive, inexpensive and simple devices in estimating overall BA levels, being conveniently employed within wineries. Furthermore, through the immobilization of a specific amine oxidase enzyme, and by crosslinking on each electrode connected in array mode, each BA could be simultaneously determined by measuring the oxidation current with an amperemeter signal [138,139]; this system gives responses in a few minutes without any pre-treatment of the samples and can be easily used. Lately, derivatization with p-toluenesulfonyl chloride has become quite a common procedure for the determination of biogenic amines in wine, as it converts poorly detectable amines into stable, UV-active sulfonamide derivatives, improving both chromatographic behavior and analytical sensitivity. Although less sensitive than fluorescent reagents, this method offers a robust and practical alternative for routine analysis using HPLC-UV systems [140,141].

**Table 4:** An overview on wine samples resulted histamine and/or tyramine free (% of total investigated samples).

Wine	Country (Region)	Samples (n)	Method	HIS Free Samples (%)	TYR Free Samples (%)	Reference
	China (Beijing, Hebei, Shandong, Tianjin, Xinjiang)	38	HPLC-FL	42%	42%	[142]
	Europe (Italy, France, Austria Germany, Spain, Switzerland)	105	HPLC-FL	16%	16%	[135]
	Greece	45	HPLC-UV	42%	29%	[79]
	Spain (Monastrell-Alicante)	14	HPLC-FL	36%	-	[143]
Red	Italy (Sardinia)	8	HPLC-FL	38%	-	[144]
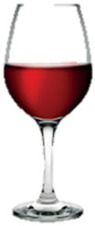	Italy (Abruzzo)	30	HPLC-UV	13%	13%	[83]
	Italy	20	HPLC-UV and LC-ESI-MS	10%	5%	[52]
	Spain (La Rioja, Madrid, Castilla-Leon, Cataluña, Castilla-La Mancha, Extremadura, Navarra)	61	HPLC-FL	25%	16%	[112]
	Croatia (Dalmatia, Hrvatsko zagorje)	24	HPLC-UV	63%	54%	[145]
	Turkey (Izmir province)	6	HPLC-FL	33%	67%	[93]
	Central Europe	35	HPLC-UV	63%	2%	[72]
	Portugal (Tejo, Dão, Douro, Lisboa, Setubal, Vinho Verde)	31	HPLC-UV	48%	65%	[146]
	Europe (France, Germany, Italy)	5	HPLC-UV	20%	40%	[118]
	Greece	47	HPLC-UV	55%	34%	[79]
	Italy (Sardinia)	7	HPLC-FL	100%	100%	[144]
White	Italy	24	HPLC-UV and LC-ESI-MS	17%	8%	[52]
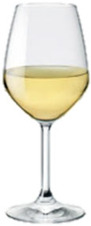	Italy (Abruzzo)	19	HPLC-UV	95%	95%	[83]
	Croatia (Dalmatia, Hrvatsko zagorje)	24	HPLC-UV	96%	92%	[145]
	Turkey (Izmir province)	4	HPLC-FL	25%	100%	[93]
	Central Europe	180	HPLC-UV	18%	8%	[72]
	Greece	8	HPLC-UV	63%	25%	[79]
Rosé	Spain	8	HPLC-UV	37%	25%	[147]
	Central Europe	17	HPLC-UV	88%	23%	[72]

## 5. Conclusions

What arises from all the studies included in this review is that the scientific community can strongly support producers in adopting mitigation strategies for BA presence in wines. In particular, for established products and respective producer countries, this will be fundamental in facing the impact of climate change, water scarcity and other unstoppable events on the final quality of wines. A lot of literature has already shown how much seasonal variability alters grapevines’ response to the content of BAs already in grape berries. A marked effect will be also seen on the environmental microbiota, which, in combination with selected starters, can shape the fermentation metabolites in musts and, further, in wines. For these reasons, while a basic achievement is to process high-hygiene and -safety raw materials, it seems that what happens in the field is little more manageable than process dynamics such as temperature maintenance, pH adjustments, correct fermentation procedures, maceration and others, as discussed. Although important pieces of information about different production systems, such as conventional vs. organic vs. biodynamic, is available, it will be important to strictly monitor these fast-changing processes, especially with respect to the use of spontaneous fermentations, the employment of new materials, longer maceration periods and different filtration procedures. An urgent message we want to clearly state is that the wine sector, like all other sectors, needs a regulatory frame, recommendations and good practices, directly spread by official bodies as EFSA. The topic of the interaction of BAs with humans is difficult to interpret, and working on this will always lead to similar conclusions, as the food and beverages sold are safe for healthy individuals. At any rate, we cannot ignore the cumulative effect of BA exposure on human health, the almost total ignorance of consumers about this risk, and the demonstrated link of allergic-like syndromes and wine consumption derived from mainly HIS and TYR action. This aspect, in our opinion, must be elucidated to producers and, especially, consumers because wine is a “cocktail” of bioactive compounds acting together, augmenting the effect of HIS. As described along with this review, not only do other BAs interact to potentiate the effect of HIS on the body, but also ethanol, sulfites and acetaldehyde contribute to exacerbating the body’s response to HIS. This association highlights the need for BA monitoring and control during winemaking to improve wine safety, particularly for consumers prone to wine intolerance and HIS sensitivity. Furthermore, we should not forget specific segments of the population treated with drugs known for lowering DAO and MAO activity, nor the role played by the simultaneous consumption of products containing variable amounts of BAs on their final accumulation per day or per meal. Lastly, the lack of strict regulatory limits for BAs in wine contributes to inconsistent monitoring and control measures across different wine producers and regions. Unlike HIS in fish, where legal limits are well-established to protect consumer health, wine does not have universally enforced BA thresholds, leading to variability in BA contents. Finally, a fair perspective would be a label claiming BAs’ absence (e.g., histamine-free), assessed by means of an official method or innovative and highly sensitive analytical procedures, to properly support the consumer in purchasing and consuming, so as to preserve health and to encourage winemakers to produce quality wines. Therefore, considering the progress in monitoring BA development in wines, it would be useful to state clearly to consumers, as a distinctive quality marker, BA-free wines.

## Figures and Tables

**Table 1:** Most common BAs, classified by the number of amine groups, and their common acronyms.

	Monoamines	Diamines	Polyamines
Biogenic amine (BA)	2-Phenylethylamine (2-PHE)	Histamine (HIS)	Spermidine (SPM)
	Tyramine (TYR)	Cadaverine (CAD)	Spermine (SPD)
	Dopamine (DOP)	Putrescine (PUT)	Tryptamine (TRYP)
		Serotonin (SER)	Agmatine (AGM)

**Table 2:** BA-related symptoms and manifestations (a); recognized conditions related to BA (b).

(a)	
BA-related symptoms	Manifestations
Respiratory	Asthma, rhinitis, general respiratory difficulties
Gastro-intestinal	Abdominal pain, nausea, diarrhea
Dermatological	Cutaneous rush, urticaria, flush
Headache	Migraines (different severity)
Heart-related symptoms	Palpitations, blood pressure disorders
(b)	
Recognized conditions	Responsible BA (mainly)
Scombroid syndrome Fish poisoning, Scombroid fish poisoning	HIS HIS
Cheese reaction Wine intolerance	TYR HIS, TYR, PUT
Booster of conditions	CAD, HIS, PUT, TYR

**Table 3:** Conditions and factors affecting the occurrence of BA in wine.

**Conditions**	**Factors**	**References**
Substrates availability (FFAs)	soil fertilization climate grape variety vineyard practice	[52,53,54,55,56,57,58]
Microbiota	*Oenococcus oeni* *Lactobacillus* spp. *Pediococcus* spp. *Enterococcus* spp. non-*Saccaromyces* yeasts *Lactobacillus* BAs degrading *Pediococcus* BAs degrading microbial multicopper oxidases	[41,47,59,60,61,62,63,64,65,66,67,68]
Chemical–physical	pH ethanol temperature	[61,62,69,70,71,72,73,74,75,76,77,78,79,80]
Winemaking process	sulfur dioxide selected yeasts spontaneous fermentations organic practice filtration clarification aging storage	[21,53,56,62,81,82,83,84,85,86,87,88,89,90,91,92,93]

## Data Availability

No new data were created or analyzed in this study. Data-sharing is not applicable to this article.

## Abbreviations

The following abbreviations are used in this manuscript:

2-PHE	2-Phenylethylamine
BAs	Biogenic amines
AGM	Agmatine
CAD	Cadaverine
DAO	Diamine Oxidase
DOP	Dopamine
EFSA	European Food Safety Authority
FAO	Food and Agriculture Organization
HIS	Histamine
MAO	Monomine Oxidase
MLF	Malolactic Fermentation
OIV	International Organization of Vine and Wine
PUT	Putrescine
SER	Serotonin
SPD	Spermine
SPM	Spermidine
TRYP	Tryptamine
TYR	Tyramine
WHO	World Health Organization
